# Association of *FOXO3* Blood DNA Methylation with Cancer Risk, Cancer Survival, and Mortality

**DOI:** 10.3390/cells10123384

**Published:** 2021-12-01

**Authors:** Chenglong Yu, Allison M. Hodge, Ee Ming Wong, Jihoon Eric Joo, Enes Makalic, Daniel Schmidt, Daniel D. Buchanan, John L. Hopper, Graham G. Giles, Melissa C. Southey, Pierre-Antoine Dugué

**Affiliations:** 1 Precision Medicine, School of Clinical Sciences at Monash Health, Monash University, Clayton, VIC 3168, Australia; chenglong.yu@monash.edu (C.Y.); ming.wong@vcgs.org.au (E.M.W.); graham.giles@cancervic.org.au (G.G.G.); melissa.southey@monash.edu (M.C.S.); 2 Cancer Epidemiology Division, Cancer Council Victoria, Melbourne, VIC 3004, Australia; allison.hodge@cancervic.org.au; 3 Centre for Epidemiology and Biostatistics, Melbourne School of Population and Global Health, The University of Melbourne, Parkville, VIC 3010, Australia; emakalic@unimelb.edu.au (E.M.); j.hopper@unimelb.edu.au (J.L.H.); 4 Department of Clinical Pathology, Melbourne Medical School, The University of Melbourne, Parkville, VIC 3010, Australia; 5 Colorectal Oncogenomics Group, Department of Clinical Pathology, Melbourne Medical School, The University of Melbourne, Parkville, VIC 3010, Australia; ji.joo@unimelb.edu.au (J.E.J.); daniel.buchanan@unimelb.edu.au (D.D.B.); 6 Victorian Comprehensive Cancer Centre, University of Melbourne Centre for Cancer Research, Parkville, VIC 3010, Australia; 7 Department of Data Science and AI, Faculty of Information Technology, Monash University, Clayton, VIC 3168, Australia; daniel.schmidt@monash.edu; 8 Genomic Medicine and Family Cancer Clinic, Royal Melbourne Hospital, Parkville, VIC 3000, Australia

**Keywords:** *FOXO3*, epigenetics, age-related outcome, longevity-related SNP, matched case–control study

## Abstract

Genetic variants in *FOXO3* are associated with longevity. Here, we assessed whether blood DNA methylation at *FOXO3* was associated with cancer risk, survival, and mortality. We used data from eight prospective case–control studies of breast (*n* = 409 cases), colorectal (*n* = 835), gastric (*n* = 170), kidney (*n* = 143), lung (*n* = 332), prostate (*n* = 869), and urothelial (*n* = 428) cancer and B-cell lymphoma (*n* = 438). Case–control pairs were matched on age, sex, country of birth, and smoking (lung cancer study). Conditional logistic regression was used to assess associations between cancer risk and methylation at 45 CpGs of *FOXO3* included on the HumanMethylation450 assay. Mixed-effects Cox models were used to estimate hazard ratios (HR) and 95% confidence intervals (CI) for associations with cancer survival (total *n* = 2286 deaths). Additionally, using data from 1088 older participants, we assessed associations of *FOXO3* methylation with overall and cause-specific mortality (*n* = 354 deaths). Methylation at a CpG in the first exon region of *FOXO3* (6:108882981) was associated with gastric cancer survival (HR = 2.39, 95% CI: 1.60–3.56, *p* = 1.9 × 10^−5^). Methylation at three CpGs in TSS1500 and gene body was associated with lung cancer survival (*p* < 6.1 × 10^−5^). We found no evidence of associations of *FOXO3* methylation with cancer risk and mortality. Our findings may contribute to understanding the implication of *FOXO3* in longevity.

## 1. Introduction

Genetic variation within the gene *FOXO3* was first shown to be strongly associated with lifespan using *C. elegans* models [1,2,3,4]. Genetic variants in human homologs of this gene were subsequently reported to be associated with longevity in several cohorts worldwide, including participants of different ancestries [5,6,7,8,9,10,11]. To our knowledge, three functional longevity-associated single nucleotide polymorphisms (SNP) in *FOXO3* have been identified: rs2802292, through its role in cellular stress response and apoptosis [12]; and rs12206094 and rs4946935, through their enhancer activity and role in mRNA expression [13]. In the largest GWAS to date [14], minor allele carriers of the variant rs2802292, together with carriers of rs10457180, which is also in *FOXO3* and in strong linkage disequilibrium (r^2^ = 0.64) with rs2802292 [15], were found to have an 8% higher and 9% lower odds, respectively, of surviving to the 90th percentile age of the population. The rs2802292 minor G-allele has been demonstrated to have protective effects on several specific age-related disorders, such as lower incidence of cancer and heart disease, in particular, coronary artery disease, and fewer bone fractures [11].

Epigenetic changes such as DNA methylation are thought to play a major role in tumourigenesis through their influence on various mechanisms, in particular, gene expression and genomic stability. DNA methylation is dependent on the one-carbon metabolism pathway, a major pathway in cancer [16], and consists of the addition of methyl groups (CH3) to cytosines at CpG dinucleotides, using DNA methyltransferases to form 5-methylcytosines (5-mC) [17]. Several studies have shown that DNA methylation in cancer-related genes measured in peripheral blood may be associated with cancer risk. Examples include hypermethylation of the *BRCA1* promoter region, which was found to be associated with increased risk of early onset breast cancer, and tumours from cases showing blood hypermethylation appear to have similar pathological features as *BRCA1* mutated tumours [18]. Blood DNA hypermethylation of an intragenic locus in *ATM*, a breast cancer predisposition gene, was also found to be associated with increased risk of breast cancer [19]. These examples highlight the relevance of constitutional DNA methylation measured in blood as indicative of a potential mechanism conferring an increased risk of disease. While *FOXO3* is not primarily known as a major cancer predisposition gene, it is considered to be a tumour suppressor due to its role in regulating the immune system, inflammation [20], and oxidative stress [21], as well as cell proliferation, DNA damage, metabolism, and apoptosis [22]. Several studies have found that dysregulation of *FOXO3* was associated with cancer progression and poor prognosis [23,24,25]. Furthermore, *FOXO3* circular RNA has also been shown to play important regulatory roles in initiation and progression of several cancer types (breast, prostate, bladder, gastric, leukemia, etc.) [26,27,28]. Alterations in DNA methylation at the *FOXO3* gene could therefore represent an additional, non-genetic mechanism affecting gene expression and causing cancer and premature death. Although the extent to which constitutional methylation changes may affect gene expression is not fully understood, there is value in assessing the association of blood DNA methylation with risk of and survival from cancer, as a major disease contributing to reduced longevity.

The aim of this study was to assess the association of DNA methylation at the *FOXO3* gene measured in peripheral blood with several age-related outcomes: cancer risk, cancer survival, and mortality.

## 2. Materials and Methods

### 2.1. Study Samples

The Melbourne Collaborative Cohort Study (MCCS) is a prospective study of 41,513 adult participants (24,469 women) aged between 27 and 76 years (99% aged 40–69) when recruited between 1990 and 1994. Southern European migrants were oversampled to extend the range of lifestyle-related exposures [29]. Participants were contacted again between 2003 and 2007 and completed similar questionnaires and physical/clinical examinations. Blood samples were taken at baseline and follow-up from 99% and 64% of participants, respectively. Baseline samples were stored as dried blood spots on Guthrie cards for the majority (73%), as peripheral blood mononuclear cell samples for 25%, and as buffy coat samples for 2% of the participants. Follow-up samples were stored as buffy coat aliquots and dried blood spots on Guthrie cards. 

Study participants provided informed consent in accordance with the Declaration of Helsinki, and the study was approved by Cancer Council Victoria’s Human Research Ethics Committee.

### 2.2. Methylation Case-Control Studies

Eight matched case–control studies nested in the MCCS were conducted to investigate the prospective association between blood DNA methylation and the risk of breast (*n* = 409 cases), colorectal (*n* = 835), gastric (*n* = 170), kidney (*n* = 143), lung (*n* = 332), prostate (*n* = 869), and urothelial (*n* = 428) cancer and mature B-cell lymphoma (*n* = 438) [30,31,32]. Cases and controls were matched on age at blood draw, sex, country of birth (Australia/New Zealand, Greece, Italy, or United Kingdom/other) and sample type (peripheral blood mononuclear cells, dried blood spots or, buffy coats) using incidence density sampling. For the lung cancer study, controls were also matched on smoking history at the time of blood collection. To minimise batch effects, samples from each matched case–control pair were plated to adjacent wells on a same slide of the assay, with plate, slide, and position of the matched pairs assigned randomly. DNA was extracted from peripheral blood taken at the time of recruitment (1990–1994), except for 151 case–control pairs of the urothelial cancer study, for which blood samples were taken at the follow-up visit in 2003–2007. The DNA source was dried blood spots, peripheral blood mononuclear cells, or buffy coats for 70%, 28%, and 2% of participants, respectively. 

### 2.3. Methylation Longitudinal Study

Methylation measures were repeated at follow-up (2003–2007) for 1100 participants who were selected as controls in the methylation case–control studies. DNA was extracted from blood samples collected on Guthrie cards at follow-up (these participants also had their baseline sample collected on a Guthrie card). After quality control, DNA methylation data were available for a subset of 1088 of the controls (Table 1). Samples collected at follow-up were used to assess associations with mortality.

### 2.4. Quality Control of DNA Methylation Data

DNA methylation was measured using the Infinium HumanMethylation450K (HM450) assay (Illumina, Inc., San Diego, CA, USA). Quality control details for processing methylation beta values have been reported previously [33]. M-values, calculated as log_2_(beta/(1-beta)), were then used for the analyses since these are thought to be more statistically valid for detection of differential methylation [34]. In this study, we focused on 45 CpGs of the *FOXO3* gene that were available on the HM450 assay (chromosome 6, hg19 positions 108,881,028 to 109,005,977). We used the Illumina annotation file to classify CpGs by functional regions (TSS1500, TSS200, 5′UTR, 1st exon, gene body, and 3′UTR).

### 2.5. Genetic Data

**Figure 1:**
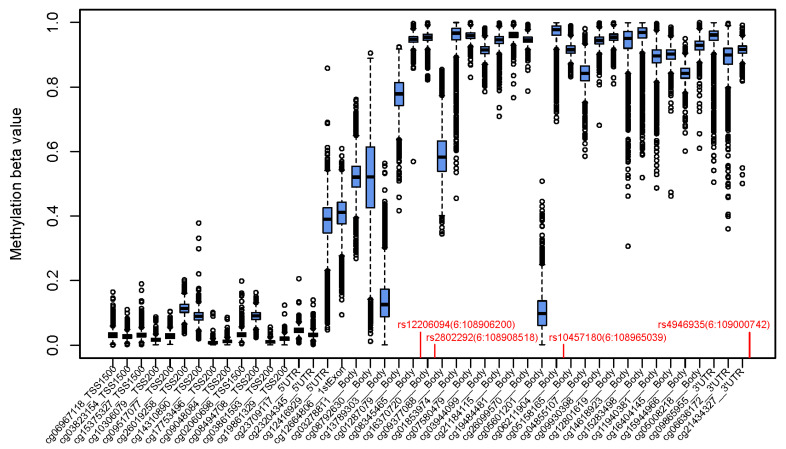
Relative position of SNPs associated with longevity and CpGs available on the HM450 assay (and their distributions of methylation values [boxplots showing median, interquartile range, and range] of 3481 control participants) of the *FOXO3* gene.

We also included genetic data for four longevity-related SNPs in *FOXO3* (rs12206094, rs2802292, rs10457180 and rs4946935, Figure 1) available in 4306 MCCS participants including 2134 cancer cases and 2172 controls, after quality controls and exclusion of genetically related participants. Genotypes were obtained using the Infinium OncoArray-500K BeadChip (Illumina, San Diego, CA, USA) [29,35] and imputed using the Michigan imputation server [36] and IMPUTE version 2 [37] with the 1000 Genomes Project dataset (phase 3) as the reference panel. To avoid bias due to confounding by shared environment among close relatives, the 4306 participants were confirmed to be unrelated, i.e., any pair among them was with a genetic relationship >0.05 (4th degree or closer relationship) [38]. 

### 2.6. Vital Status

Vital status up to 31 December 2019 was ascertained through record linkage to the Victorian Registry of Births, Deaths, and Marriages (via the Victorian Cancer Registry) and the National Death Index (via the Australian Institute of Health and Welfare). These registries were considered to be virtually complete up to 31 October 2019 and 31 August 2015 (cause of death), respectively. Causes of death were classified as follows, using the International Classification of Disease version 10: cancer: ICD-10 C00 to D49; cardiovascular disease (CVD): ICD-10 I00 to I99; other-cause: all other ICD codes, and missing (*n* = 2).

### 2.7. Statistical Analysis

#### 2.7.1. Effect of *FOXO3* SNPs on Methylation

To assess the potential influence of underlying genetic variants on our findings, we first corrected DNA methylation values using linear mixed models with M-values as the outcome, age, sex, sample type, white blood cell proportions (estimated using the Houseman algorithm [39]), and 20 genetic principal components to account for population structure as fixed effects, and study, plate, and slide of the assay as random effects, similar to a previous publication [38]. We then used linear regression to assess associations between the corrected M-values at the 45 CpGs and genetic values at the four variants. This analysis was undertaken in all 4306 samples and separately in the 2172 controls (to avoid potential collider bias). We considered associations to be significant after Bonferroni correction for multiple comparisons across four SNPs and 45 CpG sites (*p* = 0.05/(45 × 4) = 2.78 × 10^−4^).

#### 2.7.2. Cancer Risk

We assessed associations between DNA methylation at the 45 CpGs and risk of eight specific cancer types and risk of overall cancer, using conditional logistic regression models to estimate odds ratio (OR) and 95% confidence intervals (CI), expressed per standard deviation (SD) of the methylation values. In Model 1.1, we adjusted for white blood cell composition (percentage of CD4 + T cells, CD8 + T cells, B cells, NK cells, monocytes and granulocytes, estimated using the Houseman algorithm [37]) and for age and country of birth to correct for small imbalances in the matching. Sex and sample type were exactly matched between cases and controls so were not adjusted for. Model 1.2 was additionally adjusted for the main lifestyle-related factors that affect blood DNA methylation [40,41,42]: smoking status (current/former/never) and pack-years (log-transformed), alcohol consumption in the previous week (grams/day, continuous), and body mass index (BMI, continuous); matched participant pairs with missing values in any of these confounders (breast cancer: *n* = 1 pair, colorectal cancer: *n* = 17, gastric cancer: *n* = 2, kidney cancer: *n* = 2, lung cancer: *n* = 3, prostate cancer: *n* = 11, urothelial cancer: *n* = 3, mature B-cell lymphoma: *n* = 6, and overall cancer: *n* = 42) were excluded from the Model 1.2 analysis. For the overall cancer analysis, where a participant was diagnosed with more than one specific cancer type, we only included the first diagnosis (*n* = 3481), Table 1. We considered associations with risk of cancer to be significant after Bonferroni correction for multiple comparisons across nine studies and 45 CpG sites (*p* = 0.05/(45 × 9) = 1.23 × 10^−4^).

#### 2.7.3. Cancer Survival

We used mixed-effects Cox models [43] (Models 2.1 and 2.2) to estimate hazard ratios (HR) per SD for the associations between M-values at the 45 CpG sites and risk of death (all causes) following cancer diagnosis. The survival analysis was thus restricted to cancer cases. Age was used as the underlying timescale [44], and time at risk was calculated from the date of cancer diagnosis to the date of death, date of departure from Australia, or end of follow-up (31 October 2019). For the overall cancer survival analysis, where a participant was diagnosed with several cancers, we considered only their first cancer diagnosis to calculate follow-up time (*n* = 3481, among which 2185 deaths were observed). Number of deaths by cancer type was as follows: breast cancer: *n* = 160, colorectal cancer: *n* = 549, gastric cancer: *n* = 145, kidney cancer: *n* = 84, lung cancer: *n* = 312, prostate cancer: *n* = 478, urothelial cancer: *n* = 258, and mature B-cell lymphoma: *n* = 300. In Model 2.1, we adjusted for age, sex, country of birth, sample type, and white blood cell proportions as fixed effect variables and assay plate and slide as random effect variables. Model 2.2 was additionally adjusted for smoking status and pack-years, alcohol consumption, and BMI as fixed effect variables. For the overall cancer survival analysis, the models were also adjusted for cancer type (study) as a random effect variable. Cases with missing values in any of the lifestyle-related confounders (smoking status and pack-years, alcohol consumption, and BMI) were excluded from the Model 2.2 analyses: colorectal cancer: *n*= 13, kidney cancer: *n* = 1, prostate cancer: *n* = 7, urothelial cancer: *n* = 2, mature B-cell lymphoma: *n* = 4, and overall cancer: *n* = 27. We used the same *p*-value threshold as for the cancer risk analyses to detect associations (*p* = 1.23 × 10^−4^).

#### 2.7.4. Overall and Cause-Specific Mortality

We used data from the 1088 controls who had repeated methylation measures at follow-up (Table 1) to assess the association of *FOXO3* methylation with mortality. We used mixed effects Cox models to estimate hazard ratios for the association between methylation M-values and mortality using age as the underlying time scale and time at risk calculated from the date of follow-up visit to the date of death, date of departure from Australia, or end of follow-up (31 October 2019); these models were adjusted for age, sex, country of birth, sample type, and white blood cell proportions as fixed effect variables, and assay plate and slide as random effect variables (Model 3.1) and additionally for smoking status and pack-years, alcohol consumption, and BMI (Model 3.2). For the latter, participants with missing values in any of the confounders measured at follow-up were excluded (*n* = 56). In cause-specific analyses, deaths from other causes than that of interest were censored, which is a way to model the cause-specific hazard function, appropriately taking the competing risk of other-cause death into account. We considered associations to be significant using the Bonferroni correction for multiple comparisons across four outcomes and 45 CpG sites (*p* = 0.05/(45 × 4) = 2.78 × 10^−4^).

All statistical analyses were performed using R version 3.6.0.

## 3. Results

The characteristics of the MCCS samples used for investigating risk of cancer overall are presented in Table 1. Compared with controls, cases were more frequently past and current smokers and had greater smoking pack-years. The characteristics of the samples for each of the eight individual cancer case–control studies (breast, colorectal, gastric, kidney, lung, prostate, and urothelial cancers and mature B-cell lymphoma) nested within the MCCS are detailed in Table S1.

**Figure 2:**
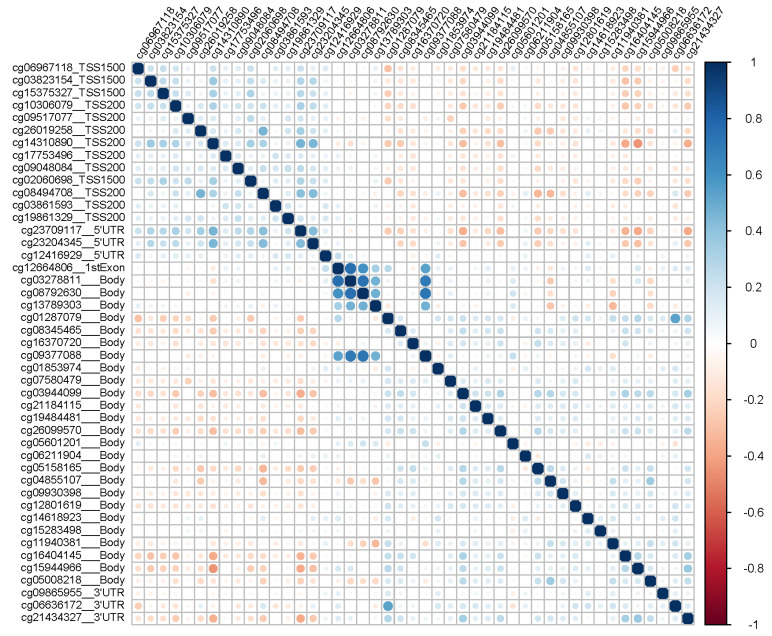
The Pearson correlation plots of methylation values of 3481 control participants at the 45 CpGs across *FOXO3*.

The positions of *FOXO3* CpGs relative to SNPs were found to be associated with longevity, and the distribution of methylation beta values of the 3481 controls across the *FOXO3* gene are shown in Figure 1. The Pearson correlation matrix of methylation values of the 3481 controls at the 45 CpGs across *FOXO3* is shown in Figure 2 and Table S2, showing generally weak correlations, typically lower than 0.3; these were null between promoter regions (TSS1500, TSS200 and 5′UTR) and the first exon, positive within the promoter and gene body regions, and negative between promoter and gene body regions. None of the four longevity-related SNPs in *FOXO3* were found to have a significant effect on any of the 45 CpGs in the region (all *p* > 2.78 × 10^−4^ and variance explained <0.4%, Table S3).

### 3.1. Associations with Cancer Risk

We found no evidence to support that methylation at any of the 45 sites in *FOXO3* was associated with risk of cancer overall or specific type (*p* > 1.23 × 10^−4^), and this finding remained the same after adjustment for lifestyle-related variables (smoking, alcohol consumption, and BMI), as in Table S4.

### 3.2. Associations with Cancer Survival

Of the 45 CpGs, we found no evidence to support an association between *FOXO3* methylation and overall cancer survival (all cancer types), and all *p* > 1.23 × 10^−4^, as in Table S5. Significant associations observed for survival from specific cancer types (*p* < 1.23 × 10^−4^) are shown in Table 2. These associations remained similar in models that additionally adjusted for lifestyle-related variables. Methylation at a CpG in the first exon region of *FOXO3* (cg12664806, 6:108882981) was associated with survival from gastric cancer (per M-value standard deviation HR = 2.39, 95% CI: 1.60–3.56, *p* = 1.9 × 10^−5^). Methylation at three CpGs was found to be associated with lung cancer survival. For these CpGs, higher methylation in the promoter region (TSS1500) was associated (*p* < 6.1 × 10^−5^) with shorter survival (cg03823154, HR = 1.33, 95% CI: 1.16–1.52) and associated with longer survival in non-promoter region (gene body) (cg05158165, HR = 0.73, 95% CI: 0.64–0.85; cg16404145, HR = 0.76, 95% CI: 0.67–0.87). No associations with survival were observed for other cancer types (*p* > 1.23 × 10^−4^, Table S5).

### 3.3. Associations with Overall Mortality

A total of 1088 participants (Table 1) were included in the overall mortality analyses, among which 354 died during follow-up. We found no evidence of an association between *FOXO3* methylation and overall or cause-specific mortality (*p* > 2.78 × 10^−4^), and this finding remained the same after adjustment for lifestyle-related variables (Table S6).

## 4. Discussion

To our knowledge, our study is the first to have comprehensively assessed the association of DNA methylation at *FOXO3* measured in blood with major health outcomes: cancer risk (3624 cases), cancer survival (2286 deaths), and mortality in an older participant sample of the MCCS (354 deaths). Our results showed strong associations between survival from cancer and DNA methylation at several CpGs. Higher methylation in the first exon region of *FOXO3* (cg12664806) was associated with shorter survival from gastric cancer. We also found that higher methylation in the promoter proximal region was associated with shorter survival from lung cancer, and in the non-promoter region with longer survival. However, no evidence of association was found at other *FOXO3* CpG sites or for other cancer types. This might imply that the regulatory role of DNA methylation is not uniform across tissues and across methylation sites, even in a very specific genomic region. Although the known functions of *FOXO3* are consistent with a potential role in cancer survival, further studies are required to elucidate the underlying mechanisms that would explain different roles of *FOXO3* methylation across cancer types and CpG sites. As we did not find any influence of *FOXO3* SNPs implicated in longevity on DNA methylation in this region, it is likely that the observed associations with survival were independent of the effects of these SNPs, and potentially act through other biological pathways. Our findings nevertheless require replication in additional studies.

Although, to our knowledge, *FOXO3* blood DNA methylation has been seldom studied, other studies of longevity-associated genes have highlighted a plausible role for DNA methylation in modulating longevity. A study by Salas-Pérez et al. [45] assessed a number of candidate genes involved in longevity-regulating pathways and found that *FOXO3* hypermethylation in leukocytes was associated, albeit weakly, with higher triglyceride levels and insulin resistance, which are risk factors for cancer and premature death; CpGs within six genes (*MTOR*, *ULK1*, *ADCY6*, *IGF1R*, *CREB5*, and *RELA*) were identified as being associated with metabolic variables. The study by Tang et al. [46] focused on the longevity gene *SIRT6* [47] in a Chinese population sample and compared the methylation profiles of 129 long-lived individuals or their immediate relatives with those of 86 individuals without a family history of exceptional longevity; they found that lower *SIRT6* promoter methylation was potentially associated with longevity. The study by Szymczak et al. [48] used whole-blood methylation profiles from 267 individuals of European ancestry (including 71 long-lived individuals) and found that methylation at *PVRL2* and *ERCC1*, genes located in the same genomic region (chromosome 19q) as the major longevity gene *APOE* [49], was associated with regulation of neurophysiological processes and cancer pathways; the authors suggested that this extended genomic region might be under both genetic and epigenetic control and be key to modulate longevity. Finally, a genome-wide methylation analysis [50] showed that differences in DNA methylation profiles between centenarians’ offspring and offspring of both non-long-lived parents predominantly occurred in key genes in aging and longevity, e.g., implicated in development, regulation of transcription, or metabolism.

The major strength of our study is its prospective design and the investigation of several cancer types and outcomes. For the cancer risk analysis, the study also involved matching of cases and controls by major cancer risk variables, which helps to control for confounding and improves statistical efficiency. Matched cases and controls were placed next to each other on a same slide of the assay, at random positions, thereby minimising batch effects. The main limitation of our study is that we did not have data to demonstrate the plausibility of blood DNA methylation at *FOXO3* as indicative of a mechanism affecting the outcomes of our study; further functional studies are therefore required to corroborate our findings. Nevertheless, the directions of the observed associations were consistent with plausible effects, because methylation in TSS and 1st exon regions are known to cause transcriptional silencing [51]. We included in the analysis all CpGs annotated to *FOXO3* by the manufacturer, but it is possible that CpGs located outside this window, or further away on chromosome 6/across the genome, e.g., through trans-genetic effects, may have functional relevance related to *FOXO3* activity. As the HM450 assay has only limited genome coverage, there may be *FOXO3* CpGs of interest that we missed, but most relevant CpGs, in particular, in promoter regions and related to cancer, were selected in priority for inclusion on the assay. Although our sample size was relatively large, we could not assess risk with precision for several sample types, in particular, risk of gastric and kidney cancer (*n* < 200). The analyses of mortality in older participants were also based on a relatively small number of deaths (*n* = 354 overall, and *n* = 60 for cancer-specific mortality). Because we used a relatively stringent strategy to account for multiple testing across outcomes and methylation sites, the *p*-value threshold to detect association was low. It is therefore possible that we could not detect existing associations due to lack of power. We were not able to further stratify the analysis by more specific causes of death, for example, dementia or individual cancer types. Large-scale studies that have measured DNA methylation are quite recent and future international pooling efforts could shed light on the potential role of *FOXO3* methylation in causing disease. In the genetic field, studies that were able to robustly identify SNPs associated with longevity did so only through the pooling of >30,000 samples and a dichotomised outcome (>90th survival percentile, compared with <60th survival percentage), and the magnitude of association was relatively modest, being estimated as an 8% increase for variant carriers for the SNP most strongly associated with longevity [14].

## 5. Conclusions

We conclude from our study that blood DNA methylation at *FOXO3* might be associated with gastric and lung cancer survival. No evidence of associations was found for other health outcomes. Additional studies based on a larger sample size are required to replicate our findings and to clarify and extend the investigation of the role of DNA methylation as a potential mechanism linking *FOXO3* to longevity.

## Figures and Tables

**Table 1:** Characteristics of the Melbourne Collaborative Cohort Study participants included in the analysis.

Participant Characteristic	Matched Case-Control Pairs		Follow-Up Sample (*n* = 1088)
	Cases of Overall Cancer (*n* = 3481)	Controls (*n* = 3481)	
Age at blood draw, median [IQR]	60.8 [54.1–65.9]	60.8 [53.9–65.6]	69.9 [62.8–75.6]
Sex:			
Male, *n* (%)	2125 (61%)	2125 (61%)	740 (68%)
Female, *n* (%)	1356 (39%)	1356 (39%)	348 (32%)
Country of birth:			
Australia/New-Zealand, *n* (%)	2380 (68%)	2367 (68%)	831 (77%)
Greece, *n* (%)	350 (10%)	361 (10%)	44 (4%)
Italy, *n* (%)	520 (15%)	520 (15%)	89 (8%)
UK/other, *n* (%)	231 (7%)	233 (7%)	124 (11%)
Blood sample type:			
Dried blood spots, *n* (%)	2412 (69%)	2412 (69%)	
Peripheral blood mononuclear cells, *n* (%)	873 (25%)	873 (25%)	1088 (100%)
Buffy coats, *n* (%)	196 (6%)	196 (6%)	
Smoking status:			
Current, *n* (%)	490 (14%)	472 (14%)	62 (6%)
Former, *n* (%)	1377 (40%)	1337 (38%)	478 (44%)
Never, *n* (%)	1613 (46%)	1671 (48%)	548 (50%)
Smoking pack-years, median [IQR]	1.6 [0–28.5]	0.75 [0–25.7]	-
Body mass index (kg/m^2^), median [IQR]	26.8 [24.4–29.6]	26.7 [24.4–29.3]	26.8 [24.2–29.4]
Alcohol consumption ^a^ (g/day), median [IQR]	4.3 [0–17.7]	4.3 [0–17.0]	8.8 [0–23.0]

^a^ Alcohol consumption was reported for the past week at baseline and in the past 12 months at follow-up.

**Table 2:** Associations between *FOXO3* methylation and cancer survival.

CpG	CHR	MAPINFO	Gene Group	Model 2.1		Model 2.2		Cancer Type
				HR (95%CI)	*p* Value	HR (95%CI)	*p* Value	
cg12664806	6	108882981	1st Exon	2.39 (1.60–3.56)	1.85 × 10^−5^	2.50 (1.67–3.74)	7.51 × 10^−6^	Gastric
cg05158165	6	108965753	Body	0.73 (0.64–0.85)	1.92 × 10^−5^	0.73 (0.63–0.84)	1.81 × 10^−5^	Lung
cg03823154	6	108879922	TSS1500	1.33 (1.16–1.52)	5.92 × 10^−5^	1.32 (1.14–1.51)	1.23 × 10^−4^	
cg16404145	6	108984834	Body	0.76 (0.67–0.87)	6.08 × 10^−5^	0.77 (0.68–0.88)	1.12 × 10^−4^	

Abbreviations: CpG: cytosine-guanine dinucleotide; CHR: chromosome; MAPINFO: CpG position (genome build 37); HR: hazard ratio, which is expressed per standard deviation of methylation M-values; CI: confidence interval. Model 2.1. Mixed effect Cox model with adjustment for age, sex, country of birth, sample type and white blood cell proportions as fixed effect variables, and assay plate and slide as random effect variables. Model 2.2. Model 2.1 + additional adjustment for smoking status and pack-years, body mass index, and alcohol consumption as fixed effect variables.

## Data Availability

Data will be made available upon reasonable request to the corresponding author.

## Supplementary Materials

The following are available online at https://www.mdpi.com/article/10.3390/cells10123384/s1. Table S1: Characteristics of the participants from eight specific studies in the MCCS sample. Table S2: Pearson correlation matrix of methylation values at the 45 *FOXO3* CpGs. Table S3: Associations between methylation values at the 45 CpGs and genetic values at the four longevity-related SNPs in *FOXO3*. Table S4: Associations between methylation values at the 45 CpGs in *FOXO3* and risk of cancer overall or specific type. Table S5: Associations between methylation values at the 45 CpGs in *FOXO3* and survival from cancer overall or specific type. Table S6: Associations between *FOXO3* methylation and overall or cause-specific mortality.
